# A Rare Type of Hepatocellular Carcinoma Presenting With Cardiac Thrombus

**DOI:** 10.7759/cureus.39611

**Published:** 2023-05-28

**Authors:** Majd B Aboona, Wendi Zhou, Karn Wijarnpreecha

**Affiliations:** 1 Internal Medicine, Department of Medicine, University of Arizona College of Medicine - Phoenix, Phoenix, USA; 2 Department of Pathology, Banner University Medical Center - Phoenix, Phoenix, USA; 3 Department of Internal Medicine, Division of Gastroenterology and Hepatology, University of Arizona College of Medicine - Phoenix, Phoenix, USA; 4 Department of Medicine, Division of Gastroenterology and Hepatology, Banner University Medical Center - Phoenix, Phoenix, USA

**Keywords:** non alcoholic fatty liver, hepatology, cirrhosis, hepatocellular carcinoma, right atrial thrombus

## Abstract

Hepatocellular carcinoma (HCC) is a complication of end stage liver disease. Even rarer is right atrial tumor thrombus burden due to HCC. Common metastatic sites of HCC in descending order are lung, peritoneum, and bone. We present a patient with liver cirrhosis due to nonalcoholic fatty liver disease (NAFLD) admitted due to incidental finding of right atrial thrombus on echocardiography after missing HCC surveillance for four years. Patient received a computed tomography (CT) scan that showed an inconclusive liver lesion despite two liver biopsies, and patient was incidentally found to have clear cell HCC diagnosed after right hepatectomy. Right atrial thrombus was treated with surgical thrombectomy and pathology showed necrotic HCC thrombi in right atrium with bile pigment. Due to the possibility of tumor growth with extrahepatic manifestations, screening in compensated cirrhosis is essential.

## Introduction

While rare, right atrial tumor thrombus complication in cirrhosis is a possibility. In previous autopsy series the prevalence of tumor burden in the right atrium ranges from 2.4-6.3% among hepatocellular carcinoma (HCC) patients (vs. 10-40% of portal vein thrombosis in HCC) [1-4].­­ The median survival range of HCC with intra-cardiac involvement is one to four months [5]. Common metastatic sites of HCC in descending order are lung, peritoneum and bone [6]. In the literature, there has been a total of 128 patients diagnosed with HCC who have had intracavitary cardiac involvement (ICI) [2]. The most common mechanism of ICI is direct extension and others include isolated metastasis in the right atrium, right ventricle, or left atrium [2]. Treatment options include excision of mass, systemic chemotherapy, trans arterial chemoembolization and radiation.

## Case presentation

**Figure 1:**
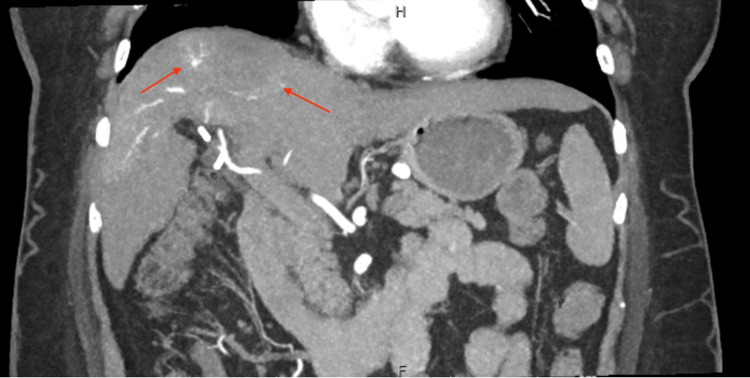
Computerized tomography (CT) scan showing a mass measuring 3.8x4.1x3.2 cm with slight peripheral nodular enhancement during the arterial phase.

**Figure 2:**
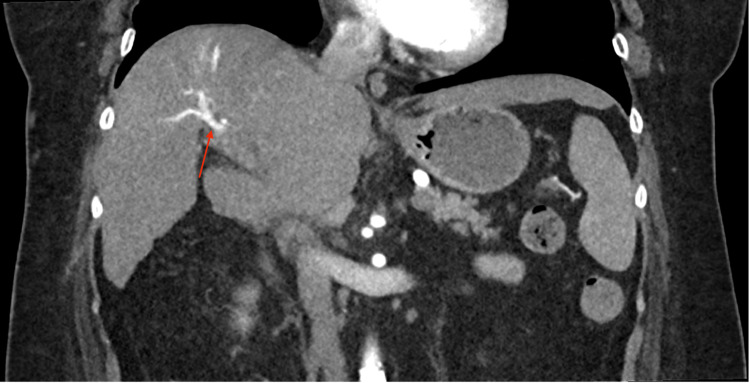
Computerized tomography (CT) scan showing thrombus extending from the liver to the heart.

A 58-year-old female with a past medical history of liver cirrhosis due to nonalcoholic fatty liver disease (NAFLD), diabetes mellitus, hypertension, and dyslipidemia who was lost to follow up for four years, was admitted from a cardiology clinic after workup for congestive heart failure due to bilateral lower extremity edema. Workup revealed incidental finding of right atrial thrombus on echocardiography. On admission, patient’s hemoglobin was 13 g/dL, platelet 166,000 K/uL, creatinine 0.97 mg/dl, sodium 145 mmol/L, albumin 4.0 g/dL, aspartate aminotransferase 48 IU/L, alkaline transaminase 31 IU/L, and alkaline phosphatase 91 IU/L. An alpha fetoprotein (AFP) was 66.2 ng/mL and cancer antigen 19-9 was 15 U/ml. Laboratory findings are summarized in Table 1. A transesophageal echocardiogram was completed which showed a large 4 cm echo density floating in the right atrium which appeared to be attached to the inferior vena cava (IVC). Patient was incidentally found to have a liver mass in the setting of compensated cirrhosis. A computerized tomography (CT) scan was obtained that showed a mass that measured 3.8 x 4.1 x 3.2 cm with slight peripheral nodular enhancement during the arterial phase (Figure 1) and a thrombus that extended from the liver to the heart (Figure 2). Interestingly, patient subsequently underwent two liver biopsies which were negative for malignancy. Multidisciplinary discussion among hepatology, surgery, radiology, and pathology department recommended surgical resection because of the complex 5 cm hepatic dome lesion despite negative liver biopsies. Patient subsequently underwent right hepatectomy. 

**Table 1:** Summary of serum analysis

Test name	Patient laboratory finding	Reference range
Hemoglobin (g/dL)	13	12-16
Platelet (K/uL)	166,000	130,000-450,000
Creatinine (mg/dL)	0.97	0.6-1.4
Sodium (mmol/L)	145	135-145
Albumin (g/dL)	4.0	3.4-4.9
Aspartate aminotransferase (U/L)	48	11-40
Alanine aminotransferase (U/L)	31	5-46
Alkaline phosphatase (U/L)	91	42-146
Alpha-fetoprotein (ng/mL)	66.2	≤ 8.7
Cancer antigen 19-9 (U/mL)	15	≤ 35

**Figure 3:**
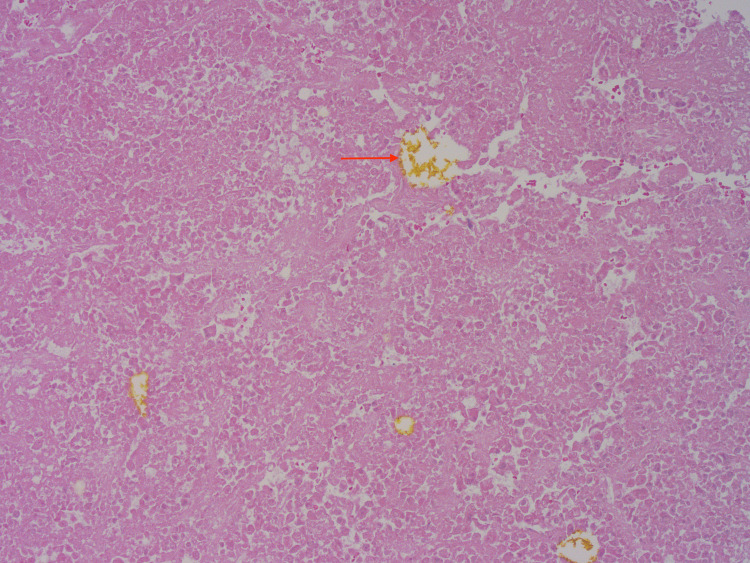
Necrotic hepatocellular carcinoma thrombi in right atrium with bile pigment. (Hematoxylin & Eosin, X 100)

**Figure 4:**
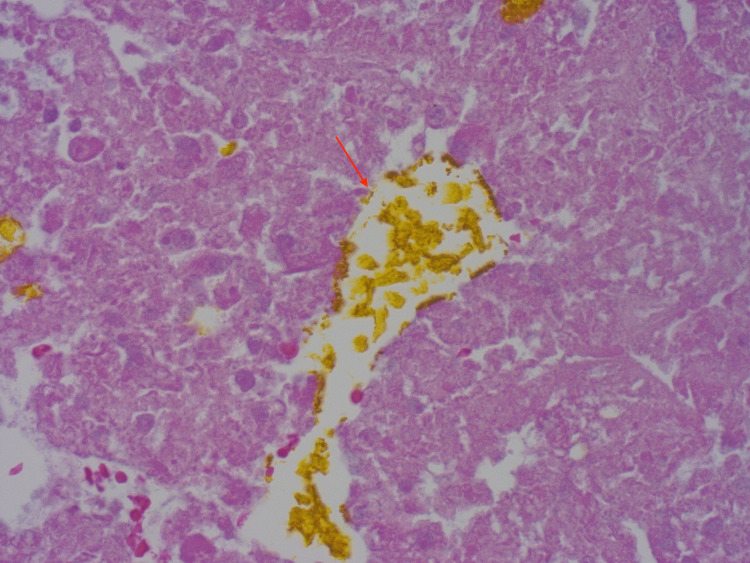
Necrotic hepatocellular carcinoma thrombi in right atrium with bile pigment. (Hematoxylin & Eosin, X 400)

Pathology from the right hepatectomy showed a poorly differentiated grade III clear cell HCC with extensive right hepatic vein invasion and presented at the vein margin. Patient’s potential right atrial thrombus was removed, and pathology showed necrotic HCC thrombi in right atrium with bile pigment (Figures 3, 4), which confirmed the etiology of cardiac thrombus from HCC. 

## Discussion

Clinical manifestation of tumor thrombus is dependent on multiple factors. Generally, if a tumor thrombus incompletely blocks the venous supply, then no clinical manifestation is present [7]. Complete obstruction of tumor thrombus can result in varicose veins in several locations such as the esophagus, gastric fundus and upper extremities [7]. Occasionally, complete tumor thrombus can result in Budd-Chiari syndrome [7]. When the heart is involved, electrocardiograms can show complete right bundle branch [7]. IVC blockage can result in low cardiac output and obstruction of the tricuspid valve. This clinical burden from HCC complications can be prevented or detected early with frequent HCC surveillance per the current guideline [8].

Diagnosis of a right atrial thrombus is obtained through different imaging modalities. A digital subtraction angiography can be obtained which will exhibit a “threads and streak sign” and “asymmetric dumbbell sign” [7]. A CT scan can be obtained which would showcase a filling defect in the invaded vascular lumen. If the tumor thrombus is through the mechanism of direct extension, then this filling defect will extend to the right atrium. A magnetic resonance imaging (MRI) can be obtained to obtain a clearer image of tumor thrombus and is typically necessary for developing a surgical plan. Echocardiography can also be useful in obtaining information regarding mobility of tumor thrombus and reliable information regarding the myocardium and valves in relation to the mass [1]. In this case a CT scan was obtained to further characterize the right atrial thrombus. However, the results of this scan unexpectedly showed a mass with enhancement in the arterial phase and suggested a tumor thrombus possibly extending from the liver (Figure 1). Despite two different operators performing the liver biopsy and two different pathologists reading the histology, HCC was not diagnosed until post-hepatectomy. These biopsies could have been negative due to needle artifact or inadequate tissue sample.

The pathophysiology of tumor burden in this case most likely originated from the IVC. A majority of right atrial invasion of HCC originates from the IVC [2]. Tumor cells are thought to penetrate the vascular endothelial cells and these cells stimulate thrombus formation [7]. Vascular invasion increases with tumor size, with AFP level >1000 ng/mL and Edmondson-Steiner grade III-IV on pathology [9]. In this case, the patient had a tumor size greater than 2 centimeters and an Edmondson-Steiner grade III of IV, increasing the risk of vascular invasion.

There are several treatment modalities for advanced HCC with tumor burden. Hepatectomy can prolong survival time in patients with tumor thrombus with studies showing median survival times of 19 months in these patients [10]. Radiation therapy has been shown to have effectiveness in stage IIIB patients, but in stage IV patients there was no significant difference in survival rates [7]. 

Treatment modalities are unfortunately limited in this advanced case of HCC. Patient’s operative course was complicated by hepatohydrothorax with submassive pulmonary embolism associated with right heart strain and left common femoral deep vein thrombus and was placed on apixaban. Localized therapies such as transarterial thromboembolization have shown to have poor therapeutic effects in these patients [7]. Atezolizumab and bevacizumab were offered to the patient given that recent updated prespecified interim analysis from the Phase 3 IMbrave050 study met primary endpoint of recurrence-free survival [11]. 

## Conclusions

This is a rare instance of essentially an incidental HCC found on imaging with a presenting clinical case of right atrial thrombus of unknown origin. Furthermore, this patient received two liver biopsies that were negative for malignancy. The diagnosis of clear cell HCC with ghost tumor cells in the right atrium was not confirmed until hepatectomy was performed. Interestingly, there is a chance of tumor growth with extrahepatic manifestations in compensated cirrhosis. This clinical burden from HCC complications could have been prevented or detected early with frequent HCC surveillance per the current guideline. This case emphasizes the importance and need for awareness of cardiac complications associated with HCC.
